# *Leishmania braziliensis* Subverts Necroptosis by Modulating RIPK3 Expression

**DOI:** 10.3389/fmicb.2018.02283

**Published:** 2018-09-28

**Authors:** Nivea F. Luz, Ricardo Khouri, Johan Van Weyenbergh, Dalila L. Zanette, Paloma P. Fiuza, Almerio Noronha, Aldina Barral, Viviane S. Boaventura, Deboraci B. Prates, Francis Ka-Ming Chan, Bruno B. Andrade, Valeria M. Borges

**Affiliations:** ^1^Instituto Gonçalo Moniz, Oswaldo Cruz Foundation (FIOCRUZ), Salvador, Brazil; ^2^Faculdade de Medicina da Bahia, Universidade Federal da Bahia, Salvador, Brazil; ^3^Department of Microbiology and Immunology, Rega Institute for Medical Research, KU Leuven, Leuven, Belgium; ^4^Departamento de Biomorfologia, Instituto de Ciências da Saúde, Universidade Federal da Bahia, Salvador, Brazil; ^5^Department of Immunology, Duke University Medical Center, Durham, NC, United States; ^6^Multinational Organization Network Sponsoring Translational and Epidemiological Research Initiative, Fundação José Silveira, Salvador, Brazil

**Keywords:** RIPK3, MLKL, macrophage, necroptosis, *Leishmania braziliensis*

## Abstract

*Leishmania braziliensis* infection causes skin ulcers, typically found in localized cutaneous leishmaniasis (LCL). This tissue pathology associates with different modalities of cell necrosis, which are subverted by the parasite as a survival strategy. Herein we examined the participation of necroptosis, a specific form of programmed necrosis, in LCL lesions and found reduced RIPK3 and PGAM5 gene expression compared to normal skin. Assays using infected macrophages demonstrated that the parasite deactivates both RIPK3 and MLKL expression and that these molecules are important to control the intracellular *L. braziliensis* replication. Thus, LCL-related necroptosis may be targeted to control infection and disease immunopathology.

## Introduction

Localized cutaneous leishmaniasis (LCL) exhibits significant disease burden in tropical regions worldwide and is characterized by one or more well-limited ulcers with raised borders. LCL caused by *Leishmania braziliensis* is associated with strong cellular responses and scarce numbers of parasites in the lesions (Scorza et al., 2017). Possible mechanisms linked to increased disease severity in LCL are still poorly understood.

Necroptosis is a form of programmed cell death that has been described to regulate key aspects of the host immune response in several infectious and non-infectious diseases (Chan et al., 2015). This cell death modality is dependent on a molecular cascade which involves sequential activation of receptor interaction protein kinase 1 (RIPK1) and RIPK3, leading to phosphorylation of mixed lineage kinase domain-like (MLKL) (Murphy et al., 2013). In addition to necroptosis, the mitochondrial phosphatase phosphoglycerate mutase family member 5 (PGAM5) also regulates certain forms of cellular necrosis (Moriwaki et al., 2016). Of note, we have recently reported that RIPK1 and PGAM5, but not RIPK3, are critical for control of *Leishmania* replication inside murine macrophages *in vitro* and *in vivo* (Farias Luz et al., 2016). Whether RIPK1–RIPK3–MLKL–PGAM5 axis is involved in human LCL is unknown.

Here, we performed an exploratory study in patients with LCL from an endemic area in Brazil, assessing *in situ* RNA expression of targets from the necroptosis pathway. In addition, *in vitro* assays using a human macrophage cell line infected with *L. braziliensis* were employed. The results identified RIPK3 and MLKL as two novel host molecules that are modulated by *Leishmania* infection and its induction may serve as potential therapeutic strategy.

## Results and Discussion

**FIGURE 1:**
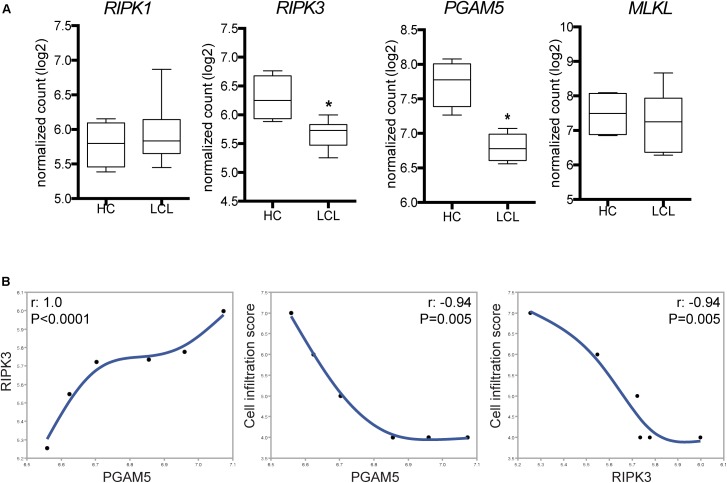
Differential expression of selected genes of necroptosis pathways in skin lesions from patients with tegumentary leishmaniasis. Total mRNA was extracted from lesion biopsy specimens obtained from six patients with localized cutaneous leishmaniasis (LCL) and normal skin samples. Indicated mRNA transcripts of host-specific cellular genes were quantified by nCounter (NanoString), including the pan-leukocyte gene CD45, for normalization of immune infiltration into tissues. **(A)** RIPK1, RIPK3, PGAM5, and MLKL expression in normal skin samples (HC) and LCL skin samples. Gene expression relative to CD45 is shown. Lines and boxes represent medians and interquartile ranges, and whiskers represent minimum and maximum values. Data were compared using the Mann–Whitney *U*-test. *^∗^P < 0.05*. **(B)** Correlation between the inflammatory cell infiltration score values and the RIPK3 and PGAM5 expression levels in skin biopsies. The Spearman rank test was used to assess correlations.

To characterize the necroptosis signaling pathway expressed *in situ* during LCL, we analyzed targeted RNA transcripts isolated from skin biopsy specimens. Patients with LCL exhibited substantial reduction in expression of *RIPK3* and *PGAM5* compared with those from normal skin (**Figure 1A**). The reduced expression of *RIPK3* found in LCL patients skin lesions suggests that *L. braziliensis* inhibits its expression to subvert necroptosis. Furthermore, no change was observed in the expression of *RIPK1* and *MLKL* between the two groups (**Figure 1A**). The normal *RIPK1* expression in LCL patients suggest that it is dispensable for disease pathogenesis, as has been observed in necroptosis induced by certain toll-like receptors and viruses (Upton et al., 2010).

We next tested whether the differences in *RIPK3* and *PGAM5* expression observed *in situ* correlated with the magnitude of cell infiltration in skin specimens. Of note, *RIPK3* and *PGAM5* expression were positively correlated with each other in LCL skin biopsies samples (**Figure 1B**). Moreover, we found that higher infiltration score correlated with lower expression of *RIPK3* and *PGAM5* in skin biopsies (**Figure 1B**). The negative correlation between expression of *RIPK3*/*PGAM5* and cellular infiltration in LCL lesion led us to hypothesize that reduced cell numbers could be due to augmented cell death in the lesions where *RIPK3*/*PGAM5* expression is the highest. RIPK3-mediated necroptosis and PGAM5-driven necrosis stimulate the release of damage-associated molecular patterns into the tissue milieu, thereby potentiating inflammation (Moriwaki and Chan, 2013; Chan et al., 2015). Thus, RIPK3 and PGAM5 could play a critical role in inducing inflammatory events at the lesion site. Because their expression negatively correlates with cellular infiltration in LCL, we speculated that *Leishmania* infection could directly inhibit RIPK3 and PGAM5 as a survival strategy. Many studies have shown *Leishmania* evasion mechanisms that enable its replication inside macrophages. These immune evasion pathways could explain *L. braziliensis* ability to inhibit RIPK3 and PGAM5. Evading RIPK3 could be beneficial for the parasite as RIPK3-driven cell death might lead to *Leishmania* clearance by at least two mechanisms: (i) preventing infected macrophages from becoming the site for parasite replication, and (ii) promoting activation of adaptive immunity via release of immunogenic danger-associated molecular patterns from dead cells.

The mitochondrial phosphatase PGAM5 participates in multiple necrotic cell death pathways. PGAM5 was first identified as a downstream RIPK3 substrate in necroptosis (Wang et al., 2012), although further studies have shown that necroptosis occurs independent of PGAM5 activation (Moriwaki et al., 2016). We cannot rule out the participation of other cell death pathways in LCL and it is possible that multiple mechanisms of cellular and tissue damage operate simultaneously in this disease. Importantly, we showed that PGAM5 promotes optimal inflammasome activation and IL-1β secretion in macrophage (Moriwaki et al., 2016). Caspase-1 and IL-1β have been described as important to controlling *Leishmania* infection in mice (Lima-Junior et al., 2013) and humans (Novais et al., 2017). Indeed, mice lacking functional RIPK1 kinase activity or PGAM5 exhibited enhanced parasite load and pronounced skin lesions when infected with various species of *Leishmania* (Farias Luz et al., 2016). These phenotypes were attributed to diminished expression of IL-1β and nitric oxide in the mutant mice (Farias Luz et al., 2016).

**FIGURE 2:**
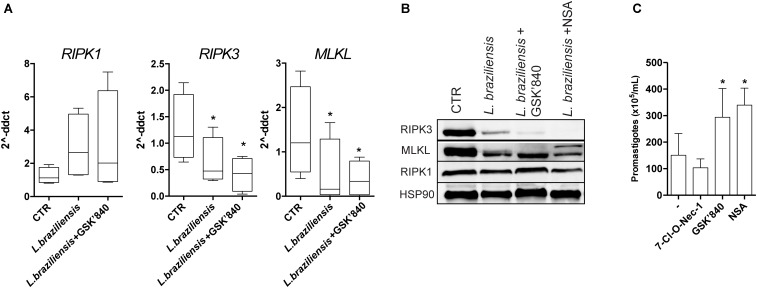
RIPK3-, but not RIPK1-mediated necroptosis, restrains *Leishmania* growth inside macrophages. PMA-differentiated THP-1 cells were pretreated with the indicated inhibitors for 1 h, followed by infection with *L. braziliensis* for 24 h. **(A)** RIPK1, RIPK3, and MLKL from macrophages was determined by real-time PCR. **(B)** RIPK1, RIPK3, and MLKL protein expression was determined by Western blot. HSP90 was used as a loading control. **(C)** PMA-differentiated THP-1 cells were pretreated with the indicated inhibitors for 1 h, followed by infection with *L. braziliensis*. The parasite load was measured by the counting of viable promastigotes as described in the Section “Patients and Methods.” Lines and boxes represent medians and interquartile ranges, and whiskers represent minimum and maximum values. Three independent experiments were performed. Values were compared by the Kruskal–Wallis test with Dunn’s multiple comparisons post-test. *^∗^P* < 0.05.

Furthermore, we asked whether the reduction in *RIPK3* mRNA expression in skin samples of LCL patients was associated to macrophages infection by *L. braziliensis*. We infected THP-1 human macrophages cell line *in vitro* and observed that *RIPK3* and *MLKL* mRNA levels were reduced following infection with *L. braziliensis*, while *RIPK* expression remained unaffected (**Figure 2A**). However, *MLKL* expression was not reduced in skin biopsies from LCL patients (**Figure 1A**). *Leishmania* is an intracellular pathogen that resides predominantly in macrophages but also in other cells including dendritic cells (Olivier et al., 2005). The differences found between our findings *ex vivo* and *in vitro* could be explained by the lower number of macrophages in the lesions with higher expression of RIPK3 and PGAM5 (**Figure 1B**). Indeed, macrophages (Farias Luz et al., 2016) and neutrophils (Arlego-Barbosa et al., 2018), are thought to be the main cell types with modified expression of necroptosis molecules in the context of *Leishmania* infection. This might explain why we found *L. braziliensis*-driven reduction of MLKL expression in macrophage cultures only (**Figures 2A,B**) and not in LCL lesion, suggesting that the other cells in the lesion are expressing very low levels of MLKL. Moreover, non-canonical necroptosis mitochondrial cell death pathways can also be inhibited in LCL lesions, such as p53-mediated necroptosis (Ranjan and Iwakuma, 2016), potentially explaining the reduced expression of PGAM5 in LCL lesions (**Figure 1A**). Together these results suggest that in the lesion, *L. braziliensis* might be the leading factor promoting RIPK3 and MLKL inhibition.

RIPK3 phosphorylates MLKL, which then multimerizes and translocates to the plasma membrane, a crucial step in necroptosis (Cai et al., 2014). Indeed, *in vitro* we found reduced protein level of both RIPK3 and MLKL in *L. braziliensis* infected macrophages (**Figure 2B**). RIPK3 inhibitor (GSK’840) has been recently described to efficiently inhibit necroptosis (Kaiser et al., 2013). As expected, THP-1 human macrophages that were pretreated with the RIPK3-specific inhibitor GSK’840 dampened both RIPK3 and MLKL mRNA (**Figure 2A**) and protein expression levels (**Figure 2B**) which reinforces the idea that MLKL mediates necroptosis signaling downstream of RIPK3. Also, as expected, macrophages treated with NSA (MLKL inhibitor) exhibited lower MLKL mRNA (**Figure 2A**) and protein expression (**Figure 2B**) values. In order to demonstrate functional effect of the compounds used in the *in vitro* experiments, cells were treated with 10 μm zVAD for 1 h, followed by TNF (100 ng/mL). Necrotic cell death was induced in this experimental setting, as detected by the decreased cell viability in macrophages treated with TNF+zVAD. As has been reported previously, necroptosis was suppressed by GSK’840, 7-Cl-O-Nec-1, and NSA (**Supplementary Figure S3**). To determine whether RIPK3 and MLKL play any role in parasite replication inside macrophages, we treated infected THP-1 cells with the different necroptosis inhibitors and evaluated parasite loads. Inhibition of RIPK3 and MLKL strongly enhanced *L. braziliensis* replication in THP-1 cells (**Figure 2C**), which was consistent with the suppressive effects of these inhibitors on RIPK3 and MLKL expression (**Figures 2A,B**). Surprisingly, the RIPK1 kinase inhibitor had no effect on *L. braziliensis* replication (**Figure 2C**). These results indicate that RIPK1 does not play a major role on *L. braziliensis* replication in macrophages, which is in contrast to infection with *L. infantum*, *L. amazonensis*, or *L. major* (Farias Luz et al., 2016). The results from our study adds to our knowledge of parasite-host interaction by indicating that *L. braziliensis* reduces RIPK3 expression in the dermis of the host to promote parasite replication. We hypothesized that the reduced expression of RIPK3 detected in skin lesion from LCL patients or *L. braziliensis* infected macrophages could be a mechanism used by the parasite to circumvent host defense. The inhibition of RIPK3, MLKL, and PGAM5 by *L. braziliensis* may be a major event associated with establishment of the parasite in the host and its replication inside macrophages.

The immunopathological mechanisms leading to necrotic cell/tissue death during LCL are not well elucidated and are likely to be multifactorial, involving other forms of inflammatory cell death besides RIPK3-dependent necroptosis and PGAM5-induced cellular necrosis. Pharmacological modulation of necroptosis in the setting of anti-*Leishmania* chemotherapy may provide consistent therapeutic benefits to patients affected by LCL. If this adjunct treatment is proven to be effective, one would expect that RIPK3 induction could increase necroptosis under conditions where apoptotic execution is inhibited. Necroptosis stimulates the immune system to elicit inflammatory responses (Chan et al., 2015). Although our study is exploratory, our data suggest that the molecular players involved in the necroptosis pathway might be useful as a target in alternative treatments in combination of immunomodulatory drugs with drugs currently used for human leishmaniasis disease. Many clinical trials studies in endemic area for leishmaniasis have pointed out the benefits of combination treatment using multiple drugs (Machado et al., 2010, 2018; Brito et al., 2014). In addition, RIPK3 inhibitors have not been used in clinical trials addressing inflammatory diseases, including leishmaniasis. Although it is speculative, it will be interesting to determine whether RIPK3 inhibitors can potentiate the efficacy of current anti-leishmanial treatments. However, further investigations are warranted to begin clinical trials to specifically target necroptosis and to elucidate the mechanisms by which RIPK3 inhibition benefits the parasite. Despite our small clinical sample size, the results from *in vitro* infection experiments further support our conclusion that RIPK3 is a critical cellular factor in LCL caused by *L. braziliensis* infection.

## Patients and Methods

This study was approved by the institutional review board from Centro de Pesquisas Gonçalo Moniz, Fundação Oswaldo Cruz, Brazil. All clinical investigations were conducted according to the Declaration of Helsinki. Written informed consent was obtained from all participants or legal guardians, and all data analyzed were de-identified.

We assessed LCL patients (*n* = six; three males and three females, mean age ± standard deviation, 37.8 ± 19.1 years), recruited at a reference clinic in Jiquiriçá, Brazil. Normal skin samples (*n* = 6), were obtained from healthy individuals by plastic surgery. Individuals included in the present study were required to be treatment-naïve and have no previous diagnosis of tegumentary leishmaniasis. A representative histopathology image of a LCL lesion is shown in **Supplementary Figure S1**. All tissue specimens were obtained before treatment; afterward, patients received *N*-methylglucamine antimoniate (20 mg/Sb/kg/d) for 15 days. LCL diagnosis was confirmed by presence of ulcerated skin lesion and one of the following: positive anti-*Leishmania* skin test or detection of parasites in skin biopsies by either histopathology (or qualitative polymerase chain reaction assays). A pathologist examined the cellular infiltration in microscopy slides using a semi-quantitative approach. Each cell-type (lymphocytes, macrophages, plasma cells, neutrophils, and eosinophils) was counted in 50 fields. The score could range from zero (absence) to 2 (>50 cells per 400× field). The total inflammatory score was calculated as the sum of scores for each cell type (**Supplementary Table S1**).

Total RNA was extracted from cryopreserved lesion biopsies using TRIzol (Invitrogen, Carlsbad, CA, United States), and purified using RNeasy columns (Qiagen, Venlo, Netherlands) as previously described (França-Costa et al., 2016). nCounter analysis (NanoString Technologies, Seattle, WA, United States) was performed based on direct molecular bar coding of target RNA transcripts and digital detection (Geiss et al., 2008). Human RIPK1, RIPK3, PGAM5, and MLKL messenger RNA (mRNA) levels were quantified *in situ*, in addition to mRNAs of several housekeeping genes (GUSB, G6PD, GAPDH, and HPRT1), for normalization, as well as leukocyte-specific genes (CD4, CD8, CD14, CD209, and CD45) (Khouri et al., 2014; França-Costa et al., 2016). Since CD45 is a pan-leukocyte marker that is expressed at both protein and transcript level in all hematopoietic subsets (T-cells, B-cells, monocytes, DCs), we have extensively tested and previously validated its use as a normalization factor to correct for differences in leukocyte skin infiltration between healthy skin and cutaneous lesions of both LCL and DCL (Khouri et al., 2014; França-Costa et al., 2016). This is clearly illustrated in **Supplementary Figure S2**, we found that higher infiltration score values positively correlated with higher expression of CD45 (**Supplementary Figure S2A**) and CD14 (**Supplementary Figure S2B**) in skin biopsies. In addition, we found positive correlation between CD14 and CD45 (**Supplementary Figure S2C**). Infiltration score was strongly and positively correlated with CD45, underscoring its use as pan-leukocyte normalization factor for differential expression.

THP-1 cells were cultured in 24-well plates and stimulated with 200 nM Phorbol 12-myristate 13-acetate (PMA) (Sigma-Aldrich, United States), for 3 days, followed by culture in medium for another 2 days. For cell viability assay necrosis was induced in THP-1 cells by pretreatment with 10 μM zVAD-fmk (R&D Systems, Minneapolis, MN, United States), 5 μM 7-Cl-O-Nec-1 (RIPK1 inhibitor); 10 μM GSK’840 (RIPK3 inhibitor) and 5 μM NSA (MLKL inhibitor) for 1 h and 100 ng/mL human TNF (R&D Systems, Minneapolis, MN, United States). Cell viability was determined 24 h post-TNF treatment by CellTiter aqueous non-radioactive cell proliferation assay (MTS) (Promega, Madison, WI, United States). *Leishmania (Viannia) braziliensis* (MHOM/BR/01/BA788) promastigotes were grown in Schneider medium (Sigma-Aldrich, United States) supplemented with 100 μ/ml penicillin, 100 mg/ml streptomycin, and 10% FBS. THP-1 cells were treated for 1 h before infection with inhibitors: 5 μM 7-Cl-O-Nec-1 (RIPK1); 10 μM GSK’840 (RIPK3) (GlaxoSmithKline) and 5 μM NSA (MLKL) (Merck Millipore’s Calbiochem^®^, Darmstadt, Germany). Cells were infected with parasites at early stationary phase [multiplicity of infection (MOI): 10]. After 4 h, cells were washed and further cultured for 24 h. Cell culture medium was then replaced by Schneider medium and the plates were kept at 24°C for 5 days, when proliferating extracellular motile promastigotes were counted in a Neubauer hemocytometer, parasite load is represented as quantity of viable Promastigotes/mL (Farias Luz et al., 2016; Quintela-Carvalho et al., 2017). Extraction of total RNA from macrophages was performed with Trizol. cDNA was synthesized using 1 μg of RNA through reverse transcription (Promega, Madison, WI, United States). The SYBR Green Mix (Applied Biosystems, Warrington, United Kingdom), 0.1–0.2 mg/mL specific primers, and 2.5 ng of cDNA were used in reactions. Expression values were calculated as delta cycle threshold (ddCt) from *GAPDH* in each sample. The primers were: 5′-GGCATTGAAGAAAAATTTAGGC-3′ and 5′-TCACAACTGCATTTTCGTTTG-3′ for *Ripk1*; 5′-GACTCCCGGCTTAGAAGGACT-3′ and 5′-CTGCTCTTGAGCTGAGACAGG-3′ for *Ripk3*; 5′-AGAGCTCCAGTGGCCATAAA-3′ and 5′-TACGCAGGATGTTGGGAGAT-3′ for *Mlkl*; and 5′-GAAATCCCATCACCATCTTCCAGG-3′ and 5′-GAGCCCCAGCCTTCTCCATG-3′ for *Gapdh*. For western blotting, whole-cell extracts were prepared from THP-1 cells using the standard 1% Nonidet P-40 lysis buffer: 150 mM NaCl, 20 mM hepes (pH 7.5), supplemented with protease, and phosphatase inhibitor cocktails (Sigma, Japan). Western blotting was performed with antibodies against RIPK1 (MAB3585, R&D Systems), RIPK3 (13526S, Cell Signaling), MLKL (194699, Abcam), and HSP90 (610419, BD Biosciences).

All the *in vitro* experiments were performed at least three times, using on average 5–6 replicates per experiment (THP-1 cells were exposed to different experimental conditions in each experiment). Comparisons between the two groups were evaluated by the Mann–Whitney *U*-test, and among multiple groups by the Kruskal–Wallis test followed by Dunn’s multiple comparisons. The Spearman rank test was used to assess correlations. *P* < 0.05 was considered significant. The Pearson test was used to assess correlations between CD14, CD45 and Cell infiltration score.

## Author Contributions

NL, RK, JVW, VSB, FC, BA, and VMB conceived and designed the study. NL, RK, DZ, and PF performed the experiments. NL, RK, JVW, DZ, VSB, DP, FC, BA, and VMB contributed with data analysis. JVW, AN, AB, VSB, and VMB provided materials and infrastructural support. NL, FC, BA, and VMB wrote and revised the manuscript.

## Conflict of Interest Statement

The authors declare that the research was conducted in the absence of any commercial or financial relationships that could be construed as a potential conflict of interest.

## Supplementary Material

FIGURE S1 Representative image of the LCL lesion. Histopathology of skin specimens obtained by biopsy of the lesion site. Intense exudative cellular reactions and presence of mononuclear cells. Digital images **(A)** 100×(lower magnification) or **(B)** 400×(higher magnification) were captured using a Nikon E600 microscope and an Olympus Q-Color digital camera with the Image Pro Plus software. **(B)** Boxed area was magnified to show inflammation marked by mononuclear cells intracellular.

FIGURE S2 Correlation between the inflammatory cell infiltration score values and CD45 and CD14 expression levels in skin lesions from patients with LCL. Total mRNA was extracted from lesion biopsy specimens obtained from six patients with localized cutaneous leishmaniasis (LCL), indicated mRNA transcripts of genes CD45 **(A)** and CD14 **(B)** were quantified by nCounter (NanoString). **(C)** Pearson correlation analysis between CD14 and CD45. A pathologist examined the cellular infiltration in microscopy as explained in the “Patients and Methods” section. Linear curve fit with 95% confidence interval were used to illustrate distribution of the data.

FIGURE S3 Functional effect of RIPK1, RIPK3 and MLKL inhibitors. THP-1 cells were treated with 10 μM zVAD-fmk, 5 μM 7-Cl-O-Nec-1 (RIPK1 inhibitor); 10 μM GSK’840 (RIPK3 inhibitor) and 5 μM NSA (MLKL inhibitor) for 1 h, followed by TNF (100 ng/mL) as indicated. Percentage of Viable Cells was determined by MTS assay as described in the Section “Patients and Methods.” Kruskal–Wallis test followed by Dunn’s multiple comparisons test were used to evaluate statistical significance. Three independent experiments were performed. Results shown are mean of quintuplicate ± SEM. *^∗^P* < 0.001.

TABLE S1 Infiltration score values from LCL skin biopsies.
