# Supplementary figures and images for: Leishmania braziliensis Subverts Necroptosis by Modulating RIPK3 Expression
Source: Front Microbiol. 2018 Sep 28;9:2283. doi: 10.3389/fmicb.2018.02283 (PMC6172319; doi:10.3389/fmicb.2018.02283)

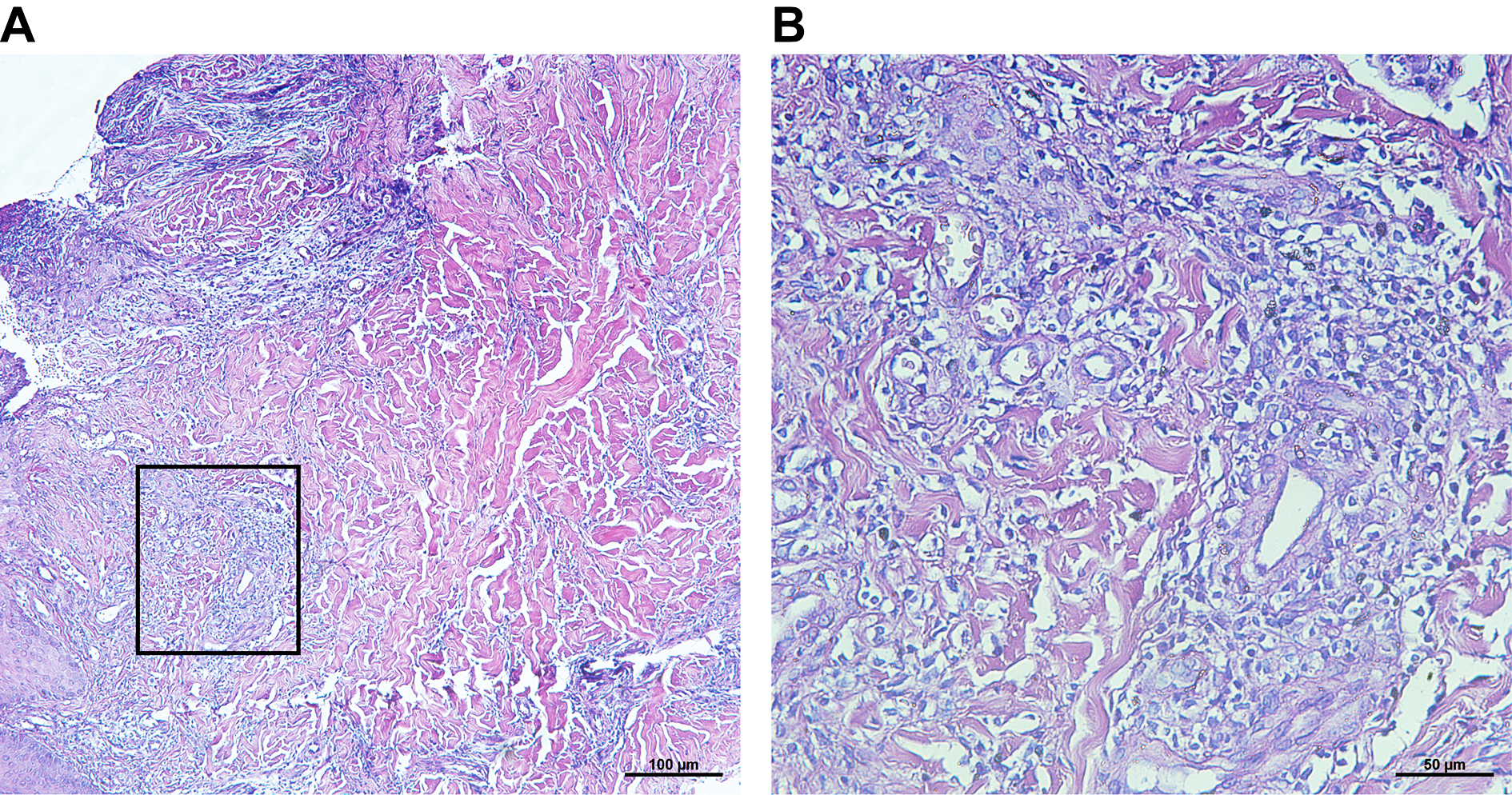

Supplement: FIGURE S1 — Representative image of the LCL lesion. Histopathology of skin specimens obtained by biopsy of the lesion site. Intense exudative cellular reactions and presence of mononuclear cells. Digital images (A) 100×(lower magnification) or (B) 400×(higher magnification) were captured using a Nikon E600 microscope and an Olympus Q-Color digital camera with the Image Pro Plus software. (B) Boxed area was magnified to show inflammation marked by mononuclear cells intracellular. [file Image_1.TIF]

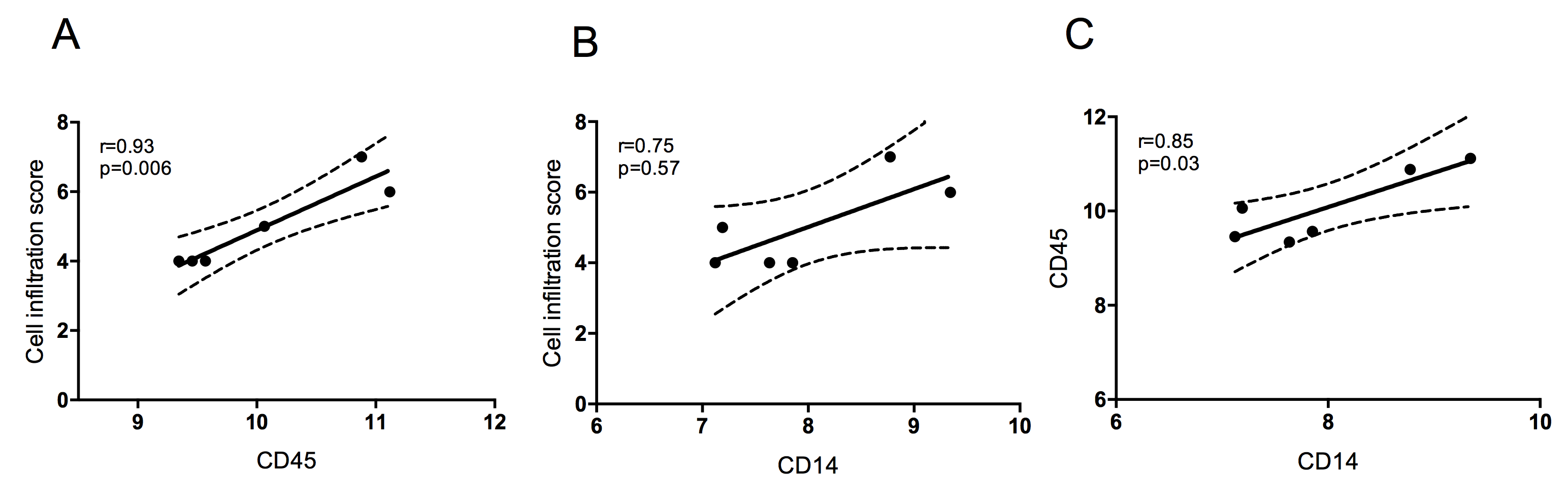

Supplement: FIGURE S2 — Correlation between the inflammatory cell infiltration score values and CD45 and CD14 expression levels in skin lesions from patients with LCL. Total mRNA was extracted from lesion biopsy specimens obtained from six patients with localized cutaneous leishmaniasis (LCL), indicated mRNA transcripts of genes CD45 (A) and CD14 (B) were quantified by nCounter (NanoString). (C) Pearson correlation analysis between CD14 and CD45. A pathologist examined the cellular infiltration in microscopy as explained in the “Patients and Methods” section. Linear curve fit with 95% confidence interval were used to illustrate distribution of the data. [file Image_2.TIFF]

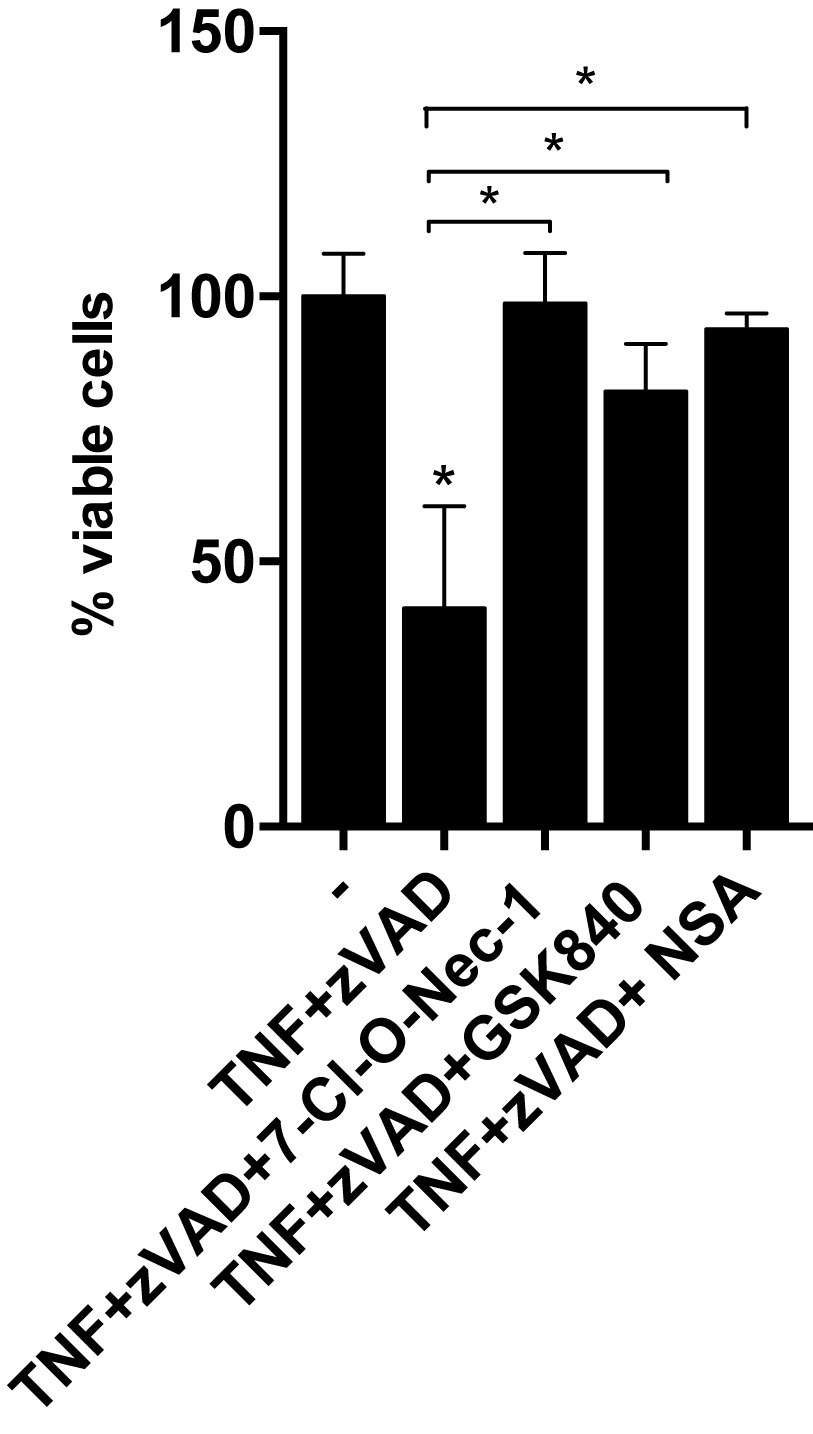

Supplement: FIGURE S3 — Functional effect of RIPK1, RIPK3 and MLKL inhibitors. THP-1 cells were treated with 10 μM zVAD-fmk, 5 μM 7-Cl-O-Nec-1 (RIPK1 inhibitor); 10 μM GSK’840 (RIPK3 inhibitor) and 5 μM NSA (MLKL inhibitor) for 1 h, followed by TNF (100 ng/mL) as indicated. Percentage of Viable Cells was determined by MTS assay as described in the Section “Patients and Methods.” Kruskal–Wallis test followed by Dunn’s multiple comparisons test were used to evaluate statistical significance. Three independent experiments were performed. Results shown are mean of quintuplicate ± SEM. ∗P < 0.001. [file Image_3.TIF]
