# Supplementary material for: Leishmania braziliensis Subverts Necroptosis by Modulating RIPK3 Expression
Source: Front Microbiol. 2018 Sep 28;9:2283. doi: 10.3389/fmicb.2018.02283 (PMC6172319; doi:10.3389/fmicb.2018.02283)
Supplement: TABLE S1 — Infiltration score values from LCL skin biopsies. [file Table_1.DOCX]

Supplementary Table 1:

|  | Lymphocytes | Eosinophils | Macrophages | Neutrophils | Plasma cells | **Inflammatory score** |
| --- | --- | --- | --- | --- | --- | --- |
| Patient #1 | 1 | 0 | 1 | 1 | 1 | **4** |
| Patient #2 | 1 | 0 | 1 | 1 | 1 | **4** |
| Patient #3 | 1 | 2 | 2 | 0 | 1 | **6** |
| Patient #4 | 1 | 2 | 2 | 0 | 0 | **5** |
| Patient #5 | 1 | 1 | 2 | 1 | 2 | **7** |
| Patient #6 | 1 | 0 | 2 | 0 | 1 | **4** |

Infiltration score values from LCL skin biopsies
